# The Up-Side of the COVID-19 Pandemic: Are Core Belief Violation and Meaning Making Associated with Post-Traumatic Growth?

**DOI:** 10.3390/ijerph20115991

**Published:** 2023-05-29

**Authors:** Marco Castiglioni, Cristina Liviana Caldiroli, Rossella Procaccia, Federica Conte, Robert A. Neimeyer, Claudia Zamin, Anna Paladino, Attà Negri

**Affiliations:** 1 Department of Human Sciences “R. Massa”, University of Milano Bicocca, 20126 Milano, Italy; marco.castiglioni@unimib.it; 2 Faculty of Psychology, eCampus University, 22060 Novedrate, Italy; rossella.procaccia@uniecampus.it; 3 Department of Psychology, University of Milano Bicocca, 20126 Milano, Italy; federica.conte@unimib.it; 4 Portland Institute for Loss and Transition, Portland, OR 97223, USA; neimeyer@portlandinstitute.org; 5 Italian Society of Relationship Psychoanalysis, 20135 Milano, Italy; 6 Department of Human and Social Sciences, University of Bergamo, 24129 Bergamo, Italyatta.negri@unibg.it (A.N.)

**Keywords:** post-traumatic growth, core beliefs violation, meaning-making, COVID-19, pandemic

## Abstract

The negative impact of the COVID-19 pandemic on mental health has been extensively documented, while its possible positive impact on the individual, defined as Post-Traumatic Growth (PTG), has been much less investigated. The present study examines the association between PTG and socio-demographic aspects, pre-pandemic psychological adjustment, stressors directly linked to COVID-19 and four psychological factors theoretically implicated in the change processes (core belief violation, meaning-making, vulnerability and mortality perception). During the second wave of the pandemic 680 medical patients completed an online survey on direct and indirect COVID-19 stressors, health and demographic information, post-traumatic growth, core belief violation, meaning-making capacity, feelings of vulnerability and perceptions of personal mortality. Violation of core beliefs, feelings of vulnerability and mortality, and pre-pandemic mental illness positively correlated with post-traumatic growth. Moreover, the diagnosis of COVID-19, stronger violation of core beliefs, greater meaning-making ability, and lower pre-existing mental illness predicted greater PTG. Finally, a moderating effect of meaning-making ability was found. The clinical implications were discussed.

## 1. Introduction

Since the outbreak of the pandemic, numerous studies have investigated the worldwide negative impact of COVID-19 on mental health [1,2,3,4,5,6,7,8,9]. Both the pandemic and the strict measures implemented for its containment (i.e., social distancing, self-isolation, stringent hygiene procedures, and lockdown) have disrupted people’s lives and well-being in nearly every nation [9,10,11,12,13,14,15]. Among the detrimental consequences are the documented increase in symptoms such as stress, anxiety, depression, psychosomatic problems, and insomnia which, on the one hand, have been found to pose serious risk factors for mental health [16,17,18,19,20,21], and on the other hand, have aggravated the already high socio-economic burden of the pandemic (e.g., rising unemployment rate and loss of productivity) [19,22]. 

However, some recent studies have focused not only on maladaptive trajectories following traumatic events but also on potentially positive psychological outcomes, a phenomenon usually identified with the construct of post-traumatic growth (PTG). Such studies have found that PTG occurred for a part of the population, suggesting that among some individuals psychological functioning has improved as a result of the pandemic, e.g., [13,23,24,25,26,27,28,29]. 

The PTG construct was first proposed by Calhoun and Tedeschi, who defined it as the positive psychological change experienced as a result of coping with intensely challenging or traumatic life events [30,31]. These authors developed a cognitive model of PTG in which they contend that the cognitive imbalance caused by a traumatic event activates a series of coping strategies, in particular, deliberative processing of the experience, which allows individuals to rebuild a positive post-traumatic understanding of the self, others, and the world, resulting in PTG [31,32,33]. Hallmarks of such growth include perceptions of increased personal strength, a greater ability to relate to others, the realization of new possibilities in life, a deepening of spiritual understanding and connection, and enhanced appreciation of life [34], culminating in the discovery or deepening of their sense of purpose and meaning in life. 

Other authors have proposed different frameworks for understanding PTG. For example, Nolen-Hoeksema and Davis [35] defined it as a coping strategy similar to positive reassessment, while Blackie et al. [36] associated PTG with possible lasting personality consequences after experiencing trauma. Some authors have even considered that, in some cases, PTG could be the manifestation of compensatory illusions [37]. These models are not mutually exclusive, as PTG, measured immediately after the onset of a crisis, could be an initial coping strategy that allows the development of long-term outcomes, such as positive and lasting changes in personality or philosophical worldview [29]. Therefore, there are different ways of understanding the concept of PTG in general and how this concept can be relevant to the analysis of the effects of the COVID-19 pandemic [29]. 

PTG is related to the construct of resilience, which indicates the complex process by which a person returns to a previous level of functioning after a traumatic or stressful event. The difference between resilience and PTG is that the latter is connected to “thriving”, which goes beyond resilience or survival in a traumatic event as it involves the search for advantages within the challenges [38]. In other words, “rather than representing growth that occurs ‘in spite of’ an adverse event, PTG is perceived by the subject as a direct consequence of having experienced an adverse event” [39] (p. 798).

Many studies have investigated the features and the processes implicated by the emergence of PTG in people who have faced adverse events, such as terrorist attacks [40], hurricanes [41], and severe acute respiratory syndrome (SARS) [42], experiencing in the aftermath significant positive life changes. Of particular relevance to the present study is the finding in studies on traumatic loss and complicated grief, that some significant degree of distress seems to be necessary to instigate PTG but not so much as to impede it. Currier et al. [43] examined the relationship between prolonged grief symptoms and PTG, finding a curvilinear association between the two: “participants who reported symptoms in an intermediate range perceived the highest levels of growth, whereas participants with relatively lower and higher levels of grief reported lower levels of growth” [43] (p. 65). These findings were closely replicated with an independent sample experiencing traumatic loss by Taku and colleagues [44].

The “optimal” balance of distress for PTG is highly subjective and depends on many predisposing factors. Several models have been elaborated to understand and explain the dynamics between complicated grief (CG) distress and PTG, some of which could be extended to other adverse events, including COVID-19. 

One such conceptualization is Hogan and Schmidt’s [45] Grief to Personal Growth Model which explains the co-occurrence of CG and PTG by positing that they spring from the same cause—shattered assumptions—a factor classically regarded by Janoff-Bulman [46] as instigating both distress and the prospect of growth from trauma in general. In this view, bereavement and other traumatic events can invalidate people’s assumptions about the benevolence of God, the person’s sense of self-worth, and the world’s controllability [46]. Such a process is called core belief violation of (CBV) by Milman and her co-investigators [19]. The struggle to come to terms with these unwelcome realities can challenge one’s personal identity and promote the conclusion that the world is meaningless and threatening after the adverse event, which can find expression in various symptomatic forms. However, the successful resolution of these challenges can also result in a deepened sense of meaning, a greater ability to relate to others, and a more stable self-concept associated with post-traumatic growth [45]. 

Similarly, Neimeyer’s Meaning Reconstruction Model [47] emphasizes the role of making meaning regarding the adverse event (i.e., the loss) and its associated distress, seeing the event narrative of the loss as a disruption in the coherence of a person’s larger self-narrative. Growth is experienced when the person is able to make meaning of the adverse event in a way that reconciles the event narrative with the self-narrative, giving it significance. Growth is further enhanced when traumatized people find some form of constructive life lesson in the experience, often revising their personal identity in light of it. We assume, based on the empirical and clinical evidence presented in the literature, including a previous study of our own [4], that similar psychological dynamics to those implicated in complicated grief were elicited by the stress factors that marked the severe phase of the COVID-19 pandemic, and that the constructs typically drawn on to explain complicated grief (CBV and disruption of meaning-making) are salient to our study participants too.

In sum, people who report having “made meaning” of a stressful life event after a CBV tend to experience fewer adverse outcomes and better adjustment, which can result in PTG. Such growth has been found to be supported by social validation of the person’s attempt to construct meaning in the experience, whereas social invalidation of such attempts has been linked to greater psychological distress [48]. 

As widely recognized, the COVID-19 pandemic generated long-lasting conditions that are highly disruptive to the processes by which people usually make sense of their lives. It posed serious challenges to personal and social belief systems on which people rely to construct a meaningful sense of their world and themselves [49] (p. 5). Milman and colleagues [19] proposed a model to explain the relationship between pandemic-related triggers and poor mental health through two above-mentioned constructs: CBV and meaning-making. They found that taking into account these two factors led to a significantly better explanation of the severity of depression, general and coronavirus-specific anxiety (i.e., specifically related to the pandemic [50]), compared to that offered by the combination of demographics and COVID-19-related stressors.

Moreover, their results showed that CBV and meaning-making partially mediated the impact of direct COVID-19 stressors (i.e., one’s own diagnosis and the death of significant others) on all mental health outcomes, and completely mediated the effects of indirect COVID-19 stressors (such as job loss and economic difficulty) on the same outcomes. 

Negri et al. [4] expanded Milman et al.’s [19] model adding two more constructs alongside CBV and meaning-making: perceived vulnerability and mortality. Their results are similar to those from the original work: a model including these four factors explains significantly more variance in mental health outcomes, compared to a model including only COVID-19 stressors and demographics. The mediation analysis also led to similar results, with the effects of COVID-19 stressors on mental health mediated partially or wholly by more severe CBV, vulnerability, and mortality and worse meaning-making.

Based on the literature regarding the association between complicated grief and PTG that found an intermediate range of grief-related symptoms as associated with higher PTG [43,44], we proposed that a correspondingly moderate violation of core beliefs due to COVID-19-related stressors would be accompanied by a meaning-making process, and associated PTG. This study, therefore, aimed to explore whether in the same Italian sample considered in Negri et al. [4], alongside the widely demonstrated negative effects, the pandemic also stimulated PTG. At the time the data for this study were collected, being diagnosed with COVID-19 or having an acquaintance who died because of COVID-19 was likely to provoke core belief violation, instigating the process of meaning reconstruction supporting PTG.

### Hypotheses

According to this perspective, we expected that:

**H1.**  *Individuals who (directly and indirectly) experienced COVID-19 stressors, apart from the well-known negative impact on their mental health, would also display a certain/significant level of PTG; as suggested in the literature reviewed above, the COVID pandemic appears to have been “tailor-made” for violating people’s core beliefs [19], hence our tentative hypothesis that COVID-19 stressors would elicit positive as well as negative responses*.

**H2.**  *Core Belief violation, vulnerability and mortality perception when associated with meaning making capacity would lead to a higher degree of PTG.*

**H3.**  *Experiencing mental health problems (i.e., anxiety, depression) before the onset of the pandemic would reduce the probability of PTG.*

**H4.**  *Following a psychological therapy during the pandemic, as a context fostering a meaning making processes, would be associated with PTG.*

## 2. Materials and Methods

### 2.1. Participants

The sample involved in this study was composed of 680 individuals between 18 and 91 years of age (M = 52.81, SD = 15.94, SE of the mean = 0.61), of whom 423 were women. Participants had to be at least 18 years old, speak fluent Italian, and provide informed consent in order to be included. The results section and Table 1 present additional demographic information. No one was excluded from the analyses and, according to our sensitivity analysis, the sample size was sufficient to reliably estimate (α = 0.05) Pearson bivariate correlations of magnitude r = 0.11 or greater with a statistical power of 0.80. Data from the same sample were used to address separate theoretical questions in a previous publication [4].

### 2.2. Procedure

We recruited participants in May and June 2021, in the northern Italian provinces of Milan and Bergamo, which were among the most affected by the pandemic breakout. Four local General Practitioners (GPs) invited all their patients to take part in the study, presented an investigation of the pandemic’s impact on psychological well-being, and provided them with a link to our online survey protocol.

All participants completed an online form at home. They signed the informed consent form in the first section and then proceeded to answer the questionnaires in a single session of approximately 30 min. The online form was set up so that respondents could not skip questions (i.e., forced response setting). All data were collected after the rigid shelter-in-place restrictions placed during the third pandemic wave had been lifted.

### 2.3. Measures

Participants completed several self-report measures evaluating their perceived post-traumatic growth as well as demographic background, experiences during the COVID-19 pandemic (i.e., from February 2020 to May 2021), violation of core beliefs, capacity for meaning-making regarding the pandemic, feelings of vulnerability and mortality, and post-pandemic mental health. The questions and questionnaires were administered in the same order listed below. Where appropriate, we describe Cronbach’s α computed in our sample alongside other measure features. Verbatim questions and answer options are reported in the Supplementary Materials.

#### 2.3.1. General and Demographic Information

In addition to the participants’ age, gender, nationality and education, we collected information on their working conditions and health (e.g., medical and psychological conditions pre-dating the COVID-19 pandemic), their marital status, the people they live with (e.g., a spouse or partner) and care for (i.e., acting as a caregiver of elderly or disabled people). 

#### 2.3.2. Stressors Associated with COVID-19

Stressors included factors directly related to the COVID-19 infection, such as being diagnosed with it or knowing someone (acquaintances or closer relations) who died because of the disease. Among the factors indirectly related to COVID-19, we considered work (i.e., job reduction/loss, working remotely or, conversely, physically going to the workplace even during lockdown), living conditions (i.e., increased costs, confinement), working with COVID-19 patients, and loss of childcare (due e.g., to schools closure).

#### 2.3.3. Psychological Factors

- *Vulnerability* was measured with a single item rated on a 6-point agreement scale (from 0 = “not at all” to 5 = “to a very high degree”): “This pandemic made me feel vulnerable and fragile”.
- *Mortality* was measured on an analogous 6-point-scale item: “This pandemic made me think more about my own death”.
- *Violation of core beliefs* assessed via the Core Beliefs Inventory (CBI) questionnaire [51,52] (Cronbach’s α = 0.90), which investigates the effects of stressful experiences on the assumptive world. The questionnaire consists of nine Likert-type items related to a range of topics (e.g., personal strengths and weaknesses, interpersonal relations, religion, and human nature). Each item is rated on a 0–5 scale (from “not at all” to “to a very great degree”) depending on how much a stressful experience has made respondents question their beliefs on that specific matter. The total score is the mean of item scores and higher values correspond to greater core belief violation.
- *Meaning making*, that is, the ability to make sense of stressful events, assessed via the Integration of Stressful Life Experiences Scale–Short Form (ISLES) [53,54] (Cronbach’s α = 0.88). The six items of the ISLES were formulated in relation to the pandemic and rated on a 1–5 scale (from “strongly agree” to “strongly disagree”). The total score was the sum of item scores and higher values correspond to better meaning-making ability.

#### 2.3.4. Mental Health and Post-Traumatic Growth Measures

- *Four-item Patient Health Questionnaire* (PHQ-4) [55,56] (Cronbach’s α = 0.86). Four items assessing depression and anxiety symptoms in a two-week span. Symptom frequency is rated 0–3 (from “not at all” to “nearly every day”), the total is the sum of item scores, and it reflects symptom severity.
- *Coronavirus Anxiety Scale* (CAS) [50,57] (Cronbach’s α = 0.86). A five-item screening tool evaluating functional impairment caused by fear and anxiety over COVID-19. The items describe situations that are rated 0–3 based on their frequency in the previous two weeks (from “not at all” to “nearly every day”). The sum of item scores represents the degree of dysfunctional Coronavirus anxiety.
- *Post-Traumatic Growth Inventory* (PTGI) [58,59] (Cronbach’s α = 0.82). A self-report scale to measure post-traumatic growth, in the Italian version. It consists of 22 items assessing perceptions of growth and positive change in response to adverse life events. The PTGI has five subscales: relationship to others (RTO), new possibilities (NP), personal strength (PS), appreciation of life (AOL) and spiritual change (SC). The items are answered on a 6-point Likert scale from 0 (no change) to 5 (complete change), with a cut-off of 61; that is, scores above 61 indicate significant PTG (moderate or high growth).
- *General Population—Clinical Outcomes in Routine Evaluation* (GP-CORE) [60,61] (Cronbach’s α = 0.83). The general population version of a self-report measure of psychological and functional wellbeing. The instrument consists of 14 items relating to the previous week and rated 0–4 based on the frequency of occurrence (from “not at all” to “most of the time”). The mean score is used as an overall indicator, with higher scores corresponding to a more compromised well-being.
- *Profile Of Mood States* (POMS) [62,63] (Cronbach’s α = 0.98). A checklist of 58 mood adjectives scored 0–4 based on how closely the respondent’s mood matched them over the previous week (from “absent” to “very much”). There are six subscales (i.e., tension, anger, fatigue, depression, confusion and vigor) and the total score, representing negative mood, was calculated by subtracting the vigor score from the sum of the other scales’ scores.

The above measures were part of a larger data collection procedure. Other measures were analyzed to answer separate theoretical questions in separate manuscripts. The Supplementary Materials and Methods section provides further details about the data collection, including a list of instruments used and not used in the present work.

### 2.4. Statistical Analyses

#### 2.4.1. Preliminary Analyses

We first computed descriptive statistics and bivariate correlations for all variables. The “marital status” variable, which was the only polytomous categorical response, was transformed to include it in the correlation analysis: participants had selected an option among four (i.e., “single”, “in a relationship but not cohabiting”, “in a relationship and cohabiting”, “divorced or widowed”) and their answer was then recoded as a set of dummy variables representing each option (e.g., single “no/yes”, in a relationship but not cohabiting “no/yes”, etc.). Descriptive statistics and correlations are briefly presented in Table 1, Table 2 and Table S1 in the Results.

Previous research has found that post-traumatic growth shares a non-linear relation with bereavement symptoms [43] An in-depth analysis of bereavement experience was beyond the scope of this work. Nonetheless, we investigated non-linear associations between PTGI and factors potentially related to bereavement, namely COVID-19 deaths (an indicator of participants’ relationship to COVID-19 victims), vulnerability and mortality. We tested three separate regression models, with PTGI as the dependent variable and each of the other three factors, in turn, as predictors. Besides the typical linear term, in each model, we included a quadratic predictor term (i.e., the squared predictor variable) to allow for the possible non-linearity of the relation. The aim of this analysis was to select suitable predictors to use in the main analysis (below), including non-linear terms if appropriate.

#### 2.4.2. Main Analysis: Predictors of Post-Traumatic Growth

Our main analysis investigated what factors significantly predicted post-traumatic growth after the pandemic. Multiple regression is well-suited to this aim because it estimates the effects of each predictor while statistically controlling for all the other variables in the model, thereby providing a comprehensive representation of phenomena. We fit a multiple regression with PTGI total score as the dependent variable and the following independent variables:

- Demographics: age, gender, education, marital status, caregiver status, number of children living in the house, diagnosed mental and physical illnesses, use of psychological or psychopharmacological therapy
- COVID-19 stressors: job loss or reduction, economic difficulties, childcare loss, confinement, working from home, leaving home to work, working with COVID-19 patients, COVID-19 diagnosis and COVID-19 deaths.
- Psychological factors: CBI, ISLES, vulnerability and mortality.
- Quadratic terms for COVID-19 deaths, vulnerability and mortality were included if they showed statistically significant effects in the preliminary analysis.

Age, COVID-19 deaths and all the psychological variables were mean-centered for this analysis.

#### 2.4.3. Exploratory Analysis: The Moderating Role of Meaning-Making

We explored the possibility that meaning-making ability could help to turn traumatic or otherwise negative experiences into a source of personal growth. Based on the results of the main analysis and on our hypotheses, we fit an additional multiple regression model, analogous to the one described in the main analysis, with the addition of two-way interaction terms involving the ISLES score. We tested whether the ISLES moderated the effects of mental illness, psychological therapy, COVID-19 diagnosis, and CBI on PTGI. Here, age, COVID-19 deaths, all the psychological variables and all the variables involved in the interactions were mean-centered.

Analyses were performed in the R environment [64]. The interaction plots in Section 3.3 were created using the package *ggeffects* [65]. 

## 3. Results

### 3.1. Preliminary Analyses

All participants provided complete answers (i.e., there were no missing data). The majority of the respondents completed post-secondary education (57%); the prevalent family arrangements were living with a partner (69.6%) and without children (60.9%) and 241 people (35.4%) met both conditions. Only 1.9% reported a mental illness (anxiety or depression), whereas approximately half of the participants had pre-existing physical illnesses (44.6%). Furthermore, 11% of respondents were involved in psychological therapy at the time of testing and 10.4% were following a psychopharmacological therapy, with 33 people (4.5% of the sample) doing both. More than half the sample (60.4%) were indirectly acquainted with a COVID-19 victim and 19.8% had lost a significant person to the infection. On the other hand, only 12.5% of respondents received a COVID-19 diagnosis themselves. See Table 1 and Table 2 for further information. 

Supplementary Table S1 shows Pearson bivariate correlations for the full set of study variables.

The three regression models we used to test for non-linear associations between PTGI and COVID-19 deaths, vulnerability and mortality showed that post-traumatic growth had a small quadratic association with COVID-19 deaths (b = −3.81, *p* = 0.035), but not with the two psychological variables (see Table S2 in Supplementary Results). Specifically, people who had known a COVID-19 victim reported a greater growth compared to those who did not know one, but it made no difference whether the victim was an acquaintance or a significant person.

### 3.2. Main Analysis: Predictors of Post-Traumatic Growth

As the results of the previous analysis showed a significant non-linear relationship between PTGI and COVID-19 deaths, we included the quadratic “COVID-19 deaths” term as a predictor in the present multiple regression.

Results, in Table 3, indicated that people who had been diagnosed with COVID-19 or experienced a stronger violation of their core beliefs (CBI) reported significantly greater post-traumatic growth: respectively, b = 5.29, *p* = 0.023 and b = 9.52, *p* < 0.001. The same was observed for participants with a greater meaning-making ability (ISLES, b = 0.40, *p* = 0.001). On the other hand, an existing mental illness was significantly associated with a smaller reported post-traumatic growth (−18.66, *p* = 0.002). Overall, the main regression model explained approximately 31% of the interindividual variance in PTGI: F (26, 652) = 12.84, *p* < 0.001 adjusted R^2^ = 0.31.

### 3.3. Exploratory Analysis: The Moderating Role of Meaning-Making

The second multiple regression model included ISLES two-way interactions with the significant PTGI predictors identified in the main analysis: mental illness, COVID-19 diagnosis and CBI. Since psychological therapy was the main object of our fourth hypothesis, we modeled an ISLES*Psychological therapy interaction to further explore the effect of this factor. Table 4 reports the analysis results.

The model accounted for 33% of PTGI variance: F (30, 648) = 11.90, *p* < 0.001, adjusted R^2^ = 0.33. Of the four interactions we tested, only two were significant: the CBI*ISLES interaction and the therapy*ISLES interaction (b = 0.25, *p* = 0.004 and b = 0.76, *p* = 0.045, respectively). 

The effect of suffering from mental illness remained quite unchanged (b = −15.13, *p* = 0.019). Receiving a positive COVID-19 diagnosis, which was the weakest among the significant predictors in the main analysis, fell below the threshold of significance in the present analysis, after controlling for ISLES interactions (b = 4.51, *p* = 0.052). 

**Figure 1:**
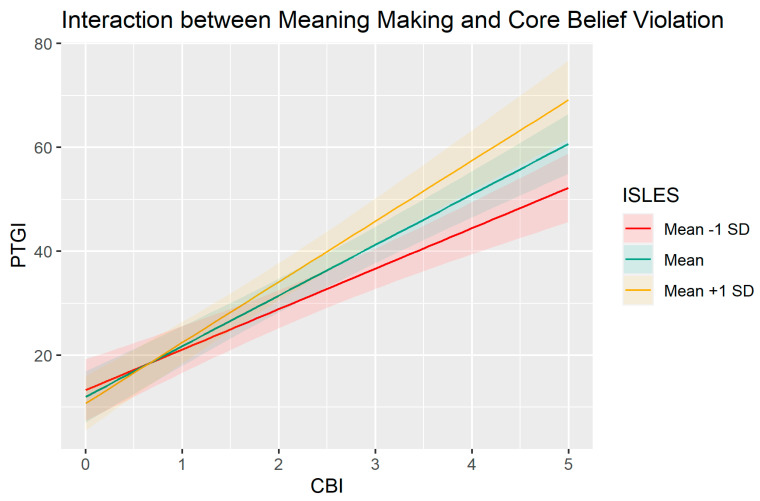
Effect of CBI on PTGI for participants with an ISLES score 1 standard deviation above, 1 standard deviation below and equal to the sample mean. PTGI = Post-traumatic growth, ISLES = Integration of Stressful Life Experiences Scale, CBI = core belief violation. Unstandardized scores.

**Figure 2:**
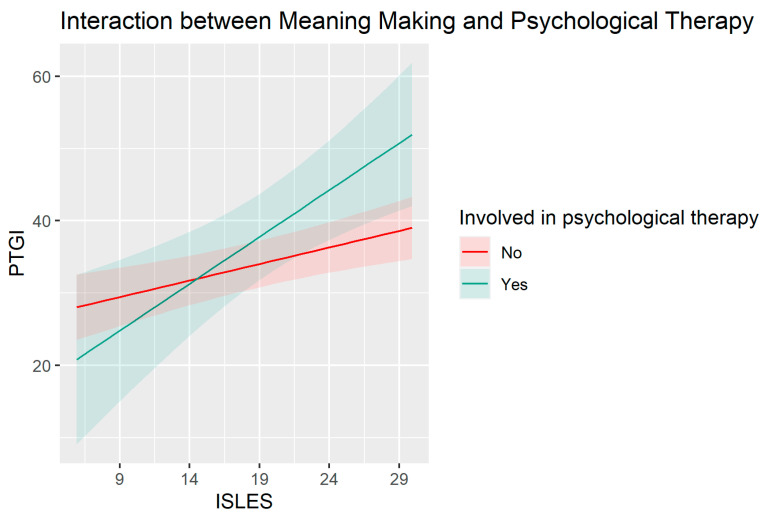
Effect of ISLES on PTGI for participants involved and not involved in psychological therapy. PTGI = Post-traumatic growth, ISLES = Integration of Stressful Life Experiences Scale. Unstandardized scores.

Looking more closely at the interactions, the positive CBI*ISLES effect (Figure 1) suggests that the association between core belief violation and post-traumatic growth is stronger for people with good meaning-making abilities. The positive psychological therapy*ISLES interaction (Figure 2) suggests that the association between meaning-making ability and post-traumatic growth is stronger for people undergoing psychological therapy.

## 4. Discussion

Since the onset of the pandemic, there has been a proliferation of studies on the adverse effects of direct and indirect COVID-19 stressors on mental health. However, relatively few studies have been concerned with analyzing positive outcomes in terms of PTG. Therefore, we aimed to test which factors are predictive of post-traumatic growth, including the following variables: demographics (sex, age, education, marital status, presence of children, and caregiving role); COVID-19 direct stressors (COVID-19 diagnosis; COVID-19 death) and indirect stressors (job loss or reduction, economic difficulties, loss of childcare, working at home, self-isolation, working with COVID-19 patients); pre- (mental illness and psychiatric illness) and post-pandemic mental health (anxiety, depression, COVID-19 anxiety, mood, and global wellbeing); psychological factors (violation of core beliefs, ability to make meaning, perception of vulnerability and mortality) and being in psychotherapy. 

The results of the preliminary analyses suggest that losing a known person is non-linearly associated with higher levels of PTG. There were differences between not having experienced a COVID-19 bereavement and having experienced a bereavement, but in contrast to previous studies, we did not find differences in the degree of affective closeness to the deceased. This finding may be explained by our focus on positive rather than negative psychological outcomes, as in studies on bereavement during the pandemic [65,66]. Although Lee and Neimeyer [66] found that closeness to the deceased in combination with unique pandemic risk factors (e.g., inability to tend to the deceased at the end of life; social isolation, traumatizing images of the loved one dying alone) contributes to dysfunctional grief, the present study suggests that confrontation with the death of an acquaintance from COVID-19, regardless of the nature of the relationship, could be enough to prompt reflective processing and personal growth. Regarding the first hypothesis concerning the effect of COVID-19-related stressors (H1), the main analyses suggest that, among the direct stressors, COVID-19 diagnosis is predictive of post-traumatic growth, whereas indirect stressors were not significant. These data mirror the results of previous studies that have identified COVID-19 infection as a stressor associated with increased physical and psychological disorders [67]. As mentioned above, Currier et al. [43] suggested that moderate stress levels activate personal adaptive resources, not only permitting the person to overcome the adverse event or return to a previous level of function but even to experience personal growth. Such stressors in our study represent a potential stimulus for the development of PTG, as opposed to indirect stressors, which, although disturbing, were found to be secondary. 

Our second hypothesis (H2) was confirmed by regression analyses. In particular, results showed that PTG is predicted by greater core belief violation and meaning-making, the two cognitive processes most related to mental health, while vulnerability and mortality had no significant predictive effects. The Milman et al. model [19] suggests that we have a set of core beliefs regarding the world as a safe place in which to confidently anticipate our future. These core beliefs maintain our sense of continuity in ourselves, our relationships, and our experiences. Traumatic events can cause psychological distress because they violate these core beliefs. They leave the individual confused and disoriented, lacking internal and external reference points. However, according to Tedeschi and Calhoun [31], perturbation in the face of traumatic events can activate resources to adapt to the situation and emerge stronger. 

Our second predictor, meaning-making, further suggests that personal growth is enhanced by the ability to develop an understanding of a stressful event and to integrate this experience into one’s belief system. However, we found that the predictive power of meaning-making as measured by the ISLES, while significant, was lower than that associated with core belief violation as measured by the CBI. While speculative, one explanation for this finding is that the assault on core meanings regarding predictability, justice, benevolence and control is a primary impetus for reviewing and revising one’s world assumptions, whereas meaning-making about one’s pandemic experience plays a secondary and supporting role in promoting growth. This suggests the importance of considering the two in combination, as in the interaction analysis discussed below.

Regarding the third hypothesis (H3): The data confirm that higher levels of prior mental illness predict lower levels of PTG. This finding is corroborated by previous studies [68,69] identifying pre-existing mental illness as a strong risk factor for poorer COVID-19 outcomes. Indeed, people with mental disorders have poorer physical health, treatment outcomes, and resilience [69,70,71]. Higher levels of psychopathology are also associated with lower levels of meaning-making, as shown in our previous study [4]. These aspects may explain the lower levels of post-infection personal growth in people with mental disorders, as the prior pathology makes these individuals more vulnerable and less able to mobilize internal and external resources to cope with the traumatic event; in other words, less able to attribute meaning to the events and integrate them in their autobiographical narrative.

Finally, our last hypothesis (H4) was only partially supported. Our data did not show a direct predictive value of being in psychotherapy on PTG. However, the results of the supplementary analyses suggested that psychological therapy significantly influences the association between meaning-making and PTG. In fact, although greater meaning-making ability is generally linked to a greater perceived PTG, this association is significantly stronger for people who were involved in psychological therapy during the pandemic. The synergistic effect of meaning-making in the context of therapy further suggests the possible value of forms of professional intervention that have sense-making regarding stressful events, and the priority of finding renewed meaning in life in their aftermath as central objectives of the therapy, supported by relevant procedures. Such goals and techniques feature prominently in meaning reconstruction therapies for loss, for example [72]. However, the finding that therapy in conjunction with meaning-making is especially salutary requires replication with a larger sample of therapy clients. 

A second significant interaction, between meaning-making and belief violation, showed that the association between belief violation and post-traumatic growth is stronger for people with good meaning-making skills. This finding is in line with previous studies on bereavement [73], which have highlighted how personal growth is linked to the subject’s ability to make sense of the traumatic event, which facilitates its integration into the self-narrative; this process restores the sense of continuity of the self-interrupted by violation of core beliefs. The moderating effect of meaning-making suggests its role in promoting growth in the wake of one’s core premises for living by restoring a measure of comprehensibility and grounding in a world made alien by loss. Ultimately, controlled trials of therapies that explicitly foster affirmative meanings in the wake of profoundly invalidating events could provide clinically relevant tests of this argument, in line with encouraging preliminary data on meaning-centered therapy for parents contending with the death of a child [74].

Some limitations should be taken into account in the interpretation of our results and may help to inform future research. Research design and enrolment are the main limitations. Specifically, the cross-sectional rather than longitudinal design cannot rule out alternative causal orders in the hypothesized relationships between psychological factors, COVID-19 stressors, preexisting disease, and PTG. A second limitation concerns the representativeness of the sample studied. It consisted of patients of general practitioners who responded to their request to complete the questionnaires. Many of them had pre-existing medical conditions for which they were being treated in general practice. Therefore, the replication of the study with different populations would increase the external validity and generalizability of the results obtained. A final limitation we highlight concerns the instruments used. They are all self-report questionnaires along with a few multiple-choice questions. Having other sources of information, such as interviews or objective measurements of subjects’ well-being, would lend greater robustness to the conclusions we drew from the results and obviate the possible biases and simplifications that online self-report data collection can entail.

## 5. Conclusions

Our data confirm the presence of post-traumatic growth processes, even in the population affected by COVID-19. The pandemic experience was classified as negative from the psychological point of view due to direct and indirect stressors related to the pandemic. However, it was also an experience capable of mobilizing individual and relational resources that led to personal growth. 

In particular, specific stressors have been found to promote higher levels of PTG. These include losing a loved one and a higher level of belief violation. All of these factors appear to promote the activation of resources that are used adaptively to emerge stronger from the adverse event. Other stressors not related to COVID-19, such as the presence of mental illness before the pandemic, were found to be associated with lower levels of PTG. It can be hypothesized that these stressors compromise the subjects’ ability to cope with the adverse event. 

There was partial support for the role of meaning-making. It had a direct association with PTG, as we expected, but its impact was lower than the violation of core beliefs. We assume that CBV—the disruption of the person’s core meanings—accounted for more of the variance in PTG, with meaning-making serving a moderating role in enhancing its effect, as the interaction effect of the two variables documents.

More generally, our results draw attention to the factors that promote personal growth in stressful contexts such as the pandemic, going beyond research focused only on its adverse outcomes. In this sense, our study could carry helpful implications for intervention in pandemic-like events to limit such events’ impact at the individual, group, social and political levels. All such levels of interventions should be aimed not only at containment measures and nonspecific psychological support but instead seek to create contexts promoting individual and community meaning-making and strengthening people’s positive core beliefs. Key to this effort would be reaffirming people’s sense of predictability, control, justice, meaningfulness, and the ability to protect those they love from deleterious outcomes. This is certainly possible in the traditional psychotherapeutic setting. However, it can also involve activating primary relational, social, and cultural contexts capable of generating collective and participatory meanings for the unexpected and often traumatic events that globalized life will increasingly lead us to experience. The main lesson learned from the COVID-19 pandemic for mental health and social workers may be precisely to review their forms of intervention to benefit the mental health of the population. In fact, becoming experts in family, community and social interventions seems to be critical to making sense of the traumatic events that can occur in this globalized world and to strengthening symbolic resources useful in protecting well-being and enhancing personal and community growth.

## Figures and Tables

**Table 1:** Participants’ distribution for demographic variables and for COVID-19 stressors.

Demographic Variable	Observed Distribution
Education	Primary school = 33 (4.8%); Secondary school = 260 (38.2%); Post-secondary school = 387 (57.0%)
Marital status	Single = 97 (14.3%); In a relationship (not living together) = 48 (7.0%); In a relationship (living together) = 473 (69.6%); Divorced/widowed = 62 (9.1%)
Children in the house	None = 414 (60.9%); One = 109 (16.0%); Two = 127 (18.7%); Three = 26 (3.8%); Four = 4 (0.6%)
Caregiver role	51 (7.5%)
Physical illness	303 (44.6%)
Mental illness	13 (1.9%)
Psychological therapy	75 (11.0%)
Psychopharmacological therapy	71 (10.4%)
COVID-19 stressors	
Job loss or reduction	336 (49.4%)
Economic difficulties	361 (53.1%)
Childcare loss	335 (49.3%)
Confinement	637 (93.7%)
Working from home	467 (68.7%)
Leaving home to work	406 (59.7%)
Work with COVID-19 patients	30 (4.4%)
COVID-19 diagnosis	85 (12.5%)
COVID-19 death	None = 134 (19.7%); Acquaintances = 411 (60.4%); Significant others = 135 (19.8%)

**Table 2:** Descriptive statistics for psychological factors, mental health and post-traumatic growth measures.

	Range	M (SD)
**Psychological factors**		
CBI	0; 5	2.26 (1.27)
ISLES	6; 30	19.08 (6.85)
Vulnerability	0; 5	2.74 (1.67)
Mortality	0; 5	2.36 (1.77)
**Mental health measures**		
PHQ	0; 12	3.97 (3.09)
CAS	0; 19	1.38 (2.71)
GP-CORE	0; 3.57	1.51 (0.6)
POMS	−29; 187	33.1 (41.35)
**Post-traumatic growth**		
PTGI-tot	0; 105	31.82 (23.10)

**Table 3:** Effects of demographic features, COVID-19 stressors and psychological factors on post-traumatic growth in linear multiple regression model.

Predictor	Estimate	SE	*p*
**(Intercept)**	**36.21**	**4.87**	**<0.001**
Gender	−0.09	1.61	0.957
Age	−0.07	0.06	0.281
Education	−0.11	1.37	0.933
MS Single ^1^	3.76	2.46	0.127
MS Relationship ^1^	0.45	3.21	0.889
MS Widowed/Divorced ^1^	4.04	2.72	0.138
Children	0.36	0.97	0.707
Caretaker role	0.98	2.88	0.734
Physical illness	−0.94	1.74	0.589
**Mental illness**	**−18.66**	**6.07**	**0.002**
Psychological therapy	3.90	2.67	0.145
Psychopharmacological therapy	−0.64	2.84	0.822
Job loss or reduction	3.93	2.98	0.188
Economic difficulties	−2.85	2.84	0.316
Childcare loss	−0.15	2.29	0.946
Working from home	0.96	2.08	0.642
Leaving home to work	−1.99	2.02	0.326
Confinement	−5.20	3.17	0.102
Work with COVID-19 patients	−1.05	3.71	0.777
**COVID-19 diagnosis**	**5.29**	**2.32**	**0.023**
COVID-19 death	1.07	1.2	0.375
COVID-19 death squared	−1.22	1.56	0.433
**CBI**	**9.52**	**0.85**	**<0.001**
**ISLES**	**0.40**	**0.12**	**0.001**
Vulnerability	0.32	0.64	0.621
Mortality	0.44	0.55	0.422

Note. Unstandardized regression coefficients. SE = Standard Error of the Estimate, MS = Marital Status, CBI = Core Beliefs Inventory, ISLES = Integration of Stressful Life Experiences Scale; ^1^ Compared to Marital Status = “In a relationship AND living together”, prevalent in the sample. Bold font = significant effect (*p* < 0.05).

**Table 4:** Effects of demographic features, COVID-19 stressors and psychological factors on post-traumatic growth in a multiple regression model with four two-way interaction terms.

Predictor	Estimate	SE	*p*
**(Intercept)**	**37.59**	**4.85**	**<0.001**
Gender	−0.06	1.59	0.970
Age	−0.06	0.06	0.381
Education	−0.15	1.36	0.910
MS Single ^1^	4.63	2.45	0.059
MS Relationship ^1^	1.11	3.19	0.728
MS Widowed/Divorced ^1^	3.87	2.70	0.152
Children	0.22	0.96	0.821
Caretaker role	1.56	2.87	0.587
Physical illness	−1.05	1.73	0.545
**Mental illness**	**−15.13**	**6.46**	**0.019**
Psychological therapy	4.34	2.66	0.103
Psychopharmacological therapy	−0.25	2.84	0.930
Job loss or reduction	3.72	2.96	0.209
Economic difficulties	−2.70	2.81	0.338
Childcare loss	0.75	2.28	0.743
Working from home	0.84	2.06	0.683
Leaving home to work	−2.03	2.01	0.313
Confinement	−5.34	3.15	0.090
Work with COVID-19 patients	−0.92	3.67	0.802
COVID-19 diagnosis	4.51	2.32	0.052
COVID-19 death	1.13	1.19	0.344
COVID-19 death squared	−1.53	1.55	0.324
**CBI**	**9.63**	**0.84**	**<0.001**
**ISLES**	**0.52**	**0.12**	**<0.001**
Vulnerability	0.46	0.64	0.477
Mortality	0.38	0.55	0.485
ISLES*Mental Illness	0.71	0.96	0.461
ISLES*COVID-19 diagnosis	−0.11	0.37	0.762
ISLES*CBI	**0.25**	**0.08**	**0.004**
ISLES*Psychological therapy	**0.76**	**0.38**	**0.045**

Note. Unstandardized regression coefficients. SE = Standard Error of the Estimate, MS = Marital Status, CBI = Core Beliefs Inventory, ISLES = Integration of Stressful Life Experiences Scale; ^1^ Compared to Marital Status = “In a relationship AND living together”, prevalent in the sample. Bold font = significant effect (*p* < 0.05).

## Data Availability

The dataset of this study is uploaded on the OSF platform. To access it, please contact the corresponding author.

## Supplementary Materials

The following supporting information can be downloaded at: https://www.mdpi.com/article/10.3390/ijerph20115991/s1, Methodological information: Note on measures collected and used, List of questions concerning demographics and COVID-19-related stressors. Table S1: Bivariate correlations. Table S2: Non-linear associations of PTGI with COVID-19 deaths, vulnerability and mortality. Figure S1: Distribution of PTGI score by type of relationship to COVID-19 victim.
