# Supplementary material for: The Up-Side of the COVID-19 Pandemic: Are Core Belief Violation and Meaning Making Associated with Post-Traumatic Growth?
Source: Int J Environ Res Public Health. 2023 May 29;20(11):5991. doi: 10.3390/ijerph20115991 (PMC10252371; doi:10.3390/ijerph20115991)
Supplement: Supplementary file 1 [file ijerph-20-05991-s001.zip › ijerph-2372709-supplementary.pdf]

Supplementary Materials

The Up-Side of the COVID-19 Pandemic: Are Core Belief Violation and Meaning Making Associated with Post-Traumatic Growth?

Marco Castiglioni [1], Cristina Liviana Caldiroli [1,*], Rossella Procaccia [2], Federica Conte [3], Robert A. Neimeyer [4], Claudia Zamin [5], Anna Paladino [6] and Attà Negri [6]

[1] Department of Human Sciences "R. Massa", University of Milano Bicocca, 20126 Milano, Italy; marco.castiglioni@unimib.it
[2] Faculty of Psychology, eCampus University, 22060 Novedrate, Italy; rossella.procaccia@uniecampus.it
[3] Department of Psychology, University of Milano Bicocca, 20126 Milano, Italy; federica.conte@unimib.it
[4] Portland Institute for Loss and Transition, Portland, OR 97223, USA; neimeyer@portlandinstitute.org
[5] Italian Society of Relationship Psychoanalysis, 20135 Milano, Italy
[6] Department of Human and Social Sciences, University of Bergamo, 24129 Bergamo, Italy; atta.negri@unibg.it
* Correspondence: cristina.caldiroli@unimib.it

Citation: Castiglioni, M.; Caldiroli, C.L.; Procaccia, R.; Conte, F.; Neimeyer, R.A.; Zamin, C.; Paladino, A.; Negri, A. The Up-Side of the COVID-19 Pandemic: Are Core Belief Violation and Meaning Making Associated with Post-Traumatic Growth? *Int. J. Environ. Res. Public Health* **2023**, *20*, 5991. https://doi.org/10.3390/ijerph20115991

Academic Editor: Lorenzo Tarsitani

Received: 14 April 2023
Revised: 23 May 2023
Accepted: 27 May 2023
Published: 29 May 2023

Copyright: © 2023 by the authors. Licensee MDPI, Basel, Switzerland. This article is an open access article distributed under the terms and conditions of the Creative Commons Attribution (CC BY) license (https://creativecommons.org/licenses/by/4.0/).

Summary

1. .... Supplementary Methods 2

1.1 Note on measures collected and used 2

1.2 Questions concerning demographics and COVID-related stressors 3

2. .... Results from the preliminary analyses 5

2.1 Bivariate correlations 5

2.2 Non-linear associations of PTGI with COVID deaths, vulnerability and mortality 8

1. Supplementary Methods

1.1 Note on measures collected and used

The data collection protocol included measures that were not used in this study. These measures will be briefly mentioned below for sake of completeness and are currently being analysed to address different theoretical questions in separate manuscripts [4]. The measures included in the analyses of the present work are fully described in the main manuscript.

Data collection protocol – instruments administered in the following fixed order:

- Questions concerning demographics and COVID-related stressors
- Vulnerability: "*This pandemic made me feel vulnerable and fragile*" answered on a 6-point scale (from 0 = "*not at all*" to 5 = "*to a very high degree*")
- Mortality: "*This pandemic made me think more about my own death*" answered on a 6-point scale (from 0 = "*not at all*" to 5 = "*to a very high degree*").
- Core Belief Inventory (CBI)
- Integration of stressful life experiences scale–short form (ISLES-SF)
- Four item Patient Health Questionnaire (PHQ-4)*
- Coronavirus Anxiety Scale (CAS)*
- Satisfaction With Life Scale (SWLS, Diener et al., 1985)*
- Post-Traumatic Growth Inventory (PTGI, Tedeschi & Calhoun, 1996)
- General Population - Clinical Outcomes in Routine Evaluation (GP-CORE)*
- Profile Of Mood States (POMS)*

* measures used to address different research questions in separate manuscripts.

1.2 Questions concerning demographics and COVID-related stressors

Participants completed the following questionnaire. Where answer options are not specified, participants were required to enter their own answer. The numbers in brackets next to answer options correspond to the value associated with that answer option in the analyses (e.g., sex was coded 0 for males and 1 for females).
Each of the stressful events listed in the last question was coded 0 if not selected and 1 if selected.

Please answer the following questions as accurately as possible:

Demographic information:

1) Age:

2) Sex:
Female (0)
Male (1)

3) Nationality:

4) Town of Residence:

5) Highest educational achievement:
Primary school (1)
Secondary school (2)
Post-secondary school (3)

6) Marital status:
Single (1)
In a relationship NOT cohabiting (2)
In a relationship and cohabiting (3)
Widowed/ Divorced (4)

8) If you have children, how many of them live with you?

7) Occupation
Student (1)
Short-term job (2)
Long-term job (3)
Unemployed and looking for job (4)
Unemployed NOT looking for job (5)
Retired (6)

9) Please, list any medical and/or psychological illnesses currently affecting you:

10) Are you currently involved in psychological therapy?
No (0)
Yes (1)

11) Are you currently involved in psychopharmacological therapy?

No (0)
Yes (1)

Questions about COVID-19 (infection, illness, hospitalization) involving you and your acquaintances

1) Have you ever been diagnosed with Coronavirus?
No (0)
Yes (1)

2) Did you know anybody who died because of Coronavirus?
No (0)
Yes, but I didn’t know them personally or directly (1)
Yes, someone significant in my life has died because of Coronavirus (2)

Stressful events related to the COVID-19 Pandemic
Please, answer the following questions using the scale provided below each one. Keep in mind that 1 corresponds to “not at all stressful” and 7 corresponds to “extremely stressful”.

1) Did the pandemic cause you stress? *
2) How stressful was the first pandemic wave (from February to May 2020)? *
3) How stressful was the second pandemic wave (from October to December 2020)? *
4) How stressful was the second pandemic wave (from February to March 2021)? *

5) What is your current level of stress due to the pandemic? *

6) Please mark from the following list all the events that you experienced during the pandemic:
- Job loss or reduction (0/1)
- Economic difficulties (0/1)
- Loss of childcare (0/1)
- Confinement (0/1)
- Working from home (0/1)
- Leaving the house for work during lockdown periods (0/1)
- Working with COVID-19 patients (0/1)
- Other (please describe)

7) What is the MOST POSITIVE event that happened to you during the pandemic? *

8) What is the MOST NEGATIVE event that happened to you during the pandemic? *

* measures used to address different research questions in separate manuscripts.

2. Results from the preliminary analyses

2.1 Bivariate correlations

Table S1 Bivariate correlations (part 1)

	Age	Gender	Education	Ms Single[a]	Ms Relationship[a]	Ms Cohabiting[a]	Ms Divorced/ Widowed[a]	Children	Caretaker Role	Physical Illness	Mental Illness
Gender	.16***										
Education	-.14***	-.03									
Ms single[a]	-.29***	-.02	.05								
Ms relationship[a]	-.27***	.01	.05	-.11**							
Ms cohabiting[a]	.24***	.03	-.02	-.62***	-.42***						
Ms divorced/ widowed[a]	.21***	-.04	-.08*	-.13***	-.09*	-.48***					
Children	-.11**	-.12**	-.01	-.25***	-.15***	.30***	-.05				
Caretaker role	.00	-.05	-.03	.17***	-.01	-.11**	-.01	-.05			
Physical Illness	.47***	.09*	-.09*	-.06	-.10*	.05	.08*	-.19***	-.02		
Mental Illness	-.03	-.06+	-.18***	.00	.00	-.02	.03	.01	.04	-.13**	
Psychological therapy	-.19***	-.11**	.02	.11**	.05	-.11**	.00	-.06	.02	-.13***	.26***
Psychopharmacological t.	-.04	-.12**	-.07+	.05	.06	-.07+	-.01	-.06	.03	.01	.37***
Job loss or reduction	-.17***	-.06	-.08*	-.04	.11**	-.06	.05	.09*	-.01	-.08*	.10*
Economic difficulties	-.12**	-.02	-.11**	-.03	.06+	-.04	.04	.11**	-.03	-.03	.09*
Childcare Loss	-.17***	-.08*	-.03	-.13***	.00	.11**	-.02	.44***	-.02	-.14***	.08*
Working from home	-.29***	-.09*	.07+	.07+	.00	-.05	.00	.15***	.06	-.17***	.09*
Leaving home to work	-.22***	-.06+	.00	.00	.00	.01	-.02	.17***	-.02	-.16***	.09*
Working with COVID patients	-.08*	.00	.10**	.04	.00	-.06	.06	.04	-.01	-.08*	-.03
Confinement	-.03	-.02	.03	.04	.00	-.05	.04	.00	-.04	.00	.04
COVID Diagnosis	-.01	-.01	.02	-.08*	-.05	.08*	.02	.12**	-.04	-.09*	.08*
COVID Death	-.01	-.05	-.01	-.01	-.03	.07+	-.07+	.05	-.02	-.01	.05
CBI	-.22***	-.24***	-.17***	.05	.03	-.07+	.03	.05	-.02	-.08*	.11**
ISLES-SF	.08*	.09*	.09*	.01	-.08+	.05	-.02	-.06	.00	.02	-.05
Vulnerability	-.16***	-.21***	-.07+	.02	.04	-.06	.04	.06	.00	-.07+	.12**
Mortality	.03	-.15***	-.04	-.03	-.04	-.02	.10**	.08*	.03	.02	.08+
CAS	.00	-.17***	-.17***	.00	-.02	-.04	.08*	.03	.04	.01	.33***
PHQ4	-.26***	-.20***	-.04	.09*	.07+	-.10*	-.01	.06	.07+	-.06+	.24***
GP-CORE	-.19***	-.11**	-.07+	.13***	.03	-.12**	.02	.00	.05	.01	.25***
POMS	-.24***	-.14***	-.04	.12**	.05	-.11**	-.01	.04	.06	-.07+	.27***

PTGI	-.18***	-.14***	-.05	.08*	.01	-.10*	.04	.03	.01	-.09*	-.03

Table S1 Bivariate correlations (part 2)

	Psychological therapy	Psychopharm acological t.	Job Loss Or Reduction	Economic Difficulties	Childcare Loss	Working from Home	Leaving Home to Work	Working with Covid Patients	Confinement	COVID Diagnosis	COVID Death
Psychopharmacological t.	.39***										
COVID Diagnosis	.07+	.06									
COVID Death	.10*	.10**	.84***								
Job loss or reduction	.01	.00	.60***	.58***							
Economic difficulties	.12**	.01	.44***	.39***	.46***						
Childcare loss	.06	.02	.54***	.50***	.55***	.51***					
Working from home	-.01	-.05	.00	-.01	.00	-.04	.10**				
Leaving home to work	.00	-.03	.04	.07+	.10**	.24***	.07+	-.06			
Working with COVID patients	-.08*	.05	.08*	.11**	.12**	.03	.09*	.01	.03		
Confinement	.06	.07+	.02	.01	-.02	.00	.00	.08*	.00	.00	
CBI	.16***	.22***	.23***	.24***	.15***	.11**	.20***	.01	.03	.11**	.09*
ISLES-SF	-.05	-.10*	-.19***	-.17***	-.19***	-.04	-.11**	.00	.05	.01	.03
Vulnerability	.14***	.21***	.10*	.11**	.10*	.10*	.10**	-.02	.02	.09*	.11**
Mortality	.04	.10**	.07+	.07+	.07+	.00	.07+	.00	.02	.09*	.14***
CAS	.17***	.28***	.11**	.12**	.10**	.02	.03	-.02	.05	.06	.13***
PHQ4	.28***	.32***	.06	.10**	.07+	.09*	.06	-.01	.01	.06	.07+
GP-CORE	.22***	.31***	.05	.10*	.05	.06	.05	-.04	.02	.05	.06
POMS	.28***	.33***	.09*	.14***	.09*	.09*	.10*	.00	.00	.05	.07+
PTGI	.12**	.09*	.12**	.11**	.06	.07+	.09*	.01	-.03	.12**	.08*

Table S1 Bivariate correlations (part 3)

	CBI	ISLES	Vulnerability	Mortality	CAS	PHQ-4	GP-CORE	POMS
ISLES-SF	-.29***							
Vulnerability	.64***	-.24***						
Mortality	.51***	-.20***	.60***					
CAS	.40***	-.19***	.35***	.37***				
PHQ4	.45***	-.17***	.49***	.30***	.46***			
GP-CORE	.43***	-.19***	.45***	.33***	.44***	.77***		
POMS	.46***	-.19***	.49***	.30***	.49***	.80***	.84***	

PTGI	.54***	-.05	.36***	.29***	.20***	.15***	.05	.12**

Note: [a] the four-level factor “Marital status” was recoded into four dichotomous variables for the analysis.

2.2 Non-linear associations of PTGI with COVID deaths, vulnerability and mortality

Table S2 Results of the quadratic regression models

	Predictor	Estimate (b)	P
Model 1			
	Intercept	33.32	.000
	COVID Death	2.91	.038
	COVID Death squared	-3.81	.035
Model 2			
	Intercept	32.55	.000
	Vulnerability	4.96	.000
	Vulnerability squared	-0.26	.425
Model 3			
	Intercept	30.29	.000
	Mortality	3.77	.000
	Mortality squared	0.49	.145

Note: Model 1= PTGI predicted by COVID Deaths (linear and quadratic term), Model 2 = PTGI predicted by Vulnerability (linear and quadratic term), Model 3 = PTGI predicted by Mortality (linear and quadratic term)

To interpret the nonlinear association between Post-traumatic growth and bereavement we plotted the distribution of PTGI score by COVID Death (Figure S1). A Kruskall-Wallis test identified significant differences in PTGI score by type of COVID-related bereavement: H (2) = 13.19, p = .001. Post-hoc tests indicated that people who did not know any COVID victim experienced a smaller post-traumatic growth compared to those who lost an acquaintance (critical difference for α = .05 was 46.78, observed difference = 70.72) or a significant person (critical difference for α = .05 was 57.35, observed difference = 59.04). Conversely, being close, rather than indirectly acquainted, to a victim did not significantly affect post-traumatic growth (critical difference for α = .05 was 46.65, observed difference = 11.67).

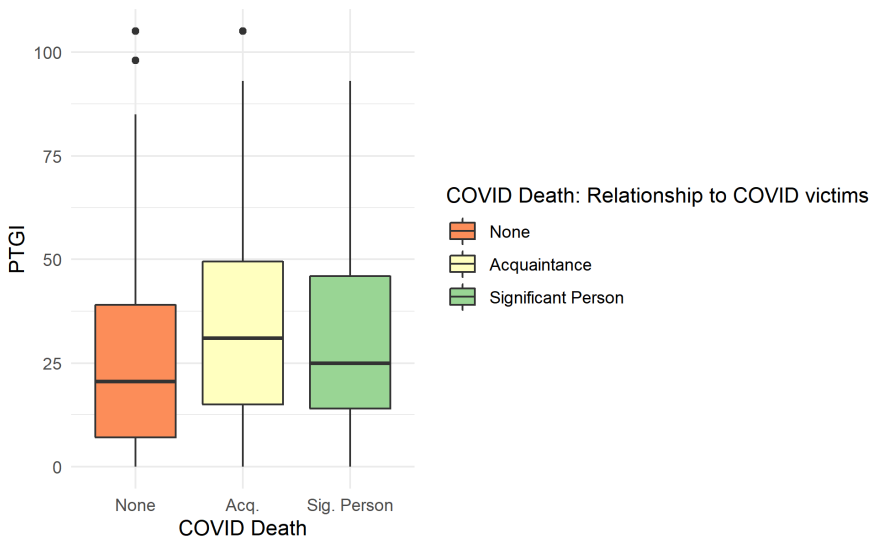

Figure S1. Distribution of PTGI score by type of relationship to COVID victim
